# Surgical Ergonomics and Musculoskeletal Pain in Orthopaedic Surgery Residents: A Multicenter Survey Study

**DOI:** 10.5435/JAAOSGlobal-D-20-00119

**Published:** 2021-03-11

**Authors:** Kade S. McQuivey, David G. Deckey, Zachary K. Christopher, Christian S. Rosenow, Lanyu Mi, Mark J. Spangehl, Joshua S. Bingham

**Affiliations:** From the Department of Orthopaedic Surgery (Dr. McQuivey, Dr. Deckey, Dr. Christopher, Ms. Mi, Dr. Spangehl, and Dr. Bingham), Mayo Clinic Arizona, Phoenix, AZ, and the Mayo Clinic Alix School of Medicine (Rosenow), Scottsdale, AZ.

## Abstract

Owing to the nature of orthopaedic surgery, occupational hazards and musculoskeletal pain (MSP) are inherent. These hazards have been well-documented among practicing orthopaedic surgeons, but there remains a paucity of data regarding MSP among orthopaedic surgery residents. The purpose of this study was to identify the prevalence and quantify the extent of work-related MSP among orthopaedic surgical residents. We also sought to analyze resident attitudes, beliefs, and behaviors regarding surgical ergonomics.

**Methods:**

An online survey was sent to 78 orthopaedic surgery resident program directors to be distributed to residents within their programs. The survey included three main sections: symptoms by body part, attitudes/beliefs/behaviors regarding surgical ergonomics, and finally demographics. Pain was reported as using the 0 to 10 Numeric Rating Scale, with 0 = no pain and 10 = maximum pain. Several questions about resident well-being were assessed using the Maslach Burnout Inventory.

**Results:**

Seventy-six orthopaedic surgery residents completed the survey, 72% men and 28% women. Most residents (97%) experience procedural-related MSP. Average pain scores of all residents was 3.52/10. Notable levels of MSP (≥4/10) were most common in the lower back (35%), neck (29.7%), and feet (25.7%). A positive association exists between higher MSP and lower work satisfaction (*P* = 0.005), burnout (*P* = 0.04), and callousness toward others (*P* < 0.0001). MSP has notable impact on resident behaviors including over-the-counter medication use, stamina, concentration, and degree of irritability.

**Conclusion:**

The prevalence of MSP among orthopaedic surgical residents is extremely high. Our study demonstrates that MSP has a notable impact on resident concentration, degree of irritability, and other burnout symptoms. The results of this study highlight the importance of limiting compromising procedural positions, ergonomic optimization, and increasing the awareness of the importance of ergonomics among residents. This could have future implications on productivity and career longevity.

Orthopaedic surgeons face a multitude of occupational hazards including exposure to smoke, toxic chemicals, needle stick injuries, infectious pathogens, radiation, auditory disturbances,^1^ and musculoskeletal pain (MSP).^2-4^ Although MSP is common among all surgical specialties, it is particularly prevalent among orthopaedic surgeons.^5^ Difficult surgical approaches requiring strenuous postures, standing for extended periods of time, and additional extremes of motion required to do surgery represent only a few of the factors that contribute to the increased rates of MSP among orthopaedic surgeons.^6^ Furthermore, orthopaedic surgery often requires considerable physical effort to manipulate and support heavy operative limbs.^7^ Although little data exist on MSP in arthroscopic orthopaedic procedures, the flexed neck and elevated arm/shoulder positions experienced during arthroscopy have been identified as a notable risk factor for MSP.^9^ Similarly, it has been demonstrated that laparoscopic surgeons experience higher rates of procedural-related pain than their colleagues who do open procedures.^8-10^

Among practicing orthopaedic surgeons, neck, back, shoulder, elbow, and wrist are the most commonly reported areas of MSP.^11,12^ Predictably, the number of years in practice and caseload have been associated with higher rates of MSP in this population.^13,14^ Although it is well-documented that practicing orthopaedic surgeons suffer high rates of MSP related to their work, a paucity of literature exists on this subject among orthopaedic surgical residents.^5,12-14^

In comparison to practicing orthopaedic surgeons, residents are generally younger, possess a smaller overall caseload, and have less cumulative exposure to occupational hazards. Although residents do not have the same caseload and number of years as practicing orthopaedic surgeons which are factors known to be notably associated with MSP,^13,14^ residents may assume the role of an assistant during surgery, which frequently requires contorted body positions for holding extremities and muscular strain with prolonged retraction, all while craning the neck attempting to visualize the surgical field. A single-institutional study found that orthopaedic surgery residents experienced similar rates of pain to that of practicing orthopaedic surgeons.^15^ This has also been demonstrated in several other subspecialties.^16,17^ Given the extensive resources allocated to training the next generation of orthopaedic providers, procedural-related injury among orthopaedic residents could represent a notable societal burden and potentially lead to burnout. Assessing the physical health of orthopaedic surgery residents may contribute to preventing cumulative chronic ailment and lost productivity through promotion of improved ergonomics and other risk-reduction strategies. The primary aim of this study was to identify the prevalence of MSP and quantify the extent of work-related MSP among orthopaedic surgery residents in the United States. Secondary aims of this study include analyzing resident behaviors, attitudes, and beliefs toward surgical ergonomics.

## Methods

This study was deemed minimal risk to participants by the institutional review board at the primary institution. The authors had direct contact information for 78 residency program directors (PDs), and thus, a web-based survey was emailed to all 78 PDs of American College of Graduate Medical Education–accredited orthopaedic surgery residencies in the United States to be distributed to residents within their respective programs. The survey included three main sections: symptoms by body part, attitudes and coping mechanisms of MSP, and demographics. Pain was reported using the 0 to 10 numerical rating scale, with 0 correlating to no pain and 10 representing maximum pain experienced.^18^ Several questions about physician well-being were assessed using the Maslach Burnout Inventory.^19^ The survey was emailed in February 2020, and residents were given a period of two months to complete the survey. The survey was closed in April 2020. Two separate electronic mailings were disbursed to residency PDs to forward to their residents to maximize response rates. A descriptive analysis was done to calculate means, percentages, ranges, SDs, and interquartile ranges (IQRs). A univariate analysis was then done with pain experience as the continuous outcome, and another with job satisfaction/callousness as the continuous variables. Statistical significance was determined using a *P* value of 0.05. Statistical analysis was done using SAS software, Version 9.4 of the SAS System for Unix.

## Results

### Demographics

A total of 76 orthopaedic surgery residents responded to the survey, 72% men and 28% women. The mean age of participants was 29.9 years old (range 26 to 39 years) with a mean postgraduation year (PGY) of 2.68 (range 1 to 5). Mean weight was 173.1 pounds (range 115 to 270 pounds), mean height was 5'10’’ (range 5'3’’ to 6'8’’) for a mean body mass index 23.74 kg/m^2^ (range 18.7 to 34.7). Only 2.7% of respondents were left handed. The average amount of time spent in the surgical room (OR) per week was 24 hr/wk (IQR 20 to 30 hr/wk). The average number of cases residents anticipate completing by the end of their residency was 2000 cases (IQR 1500 to 2050 cases). See Table 1 for the demographics of resident survey participants. No association was noted between higher pain scores and sex (*P* = 0.1), PGY year (*P* = 0.15), age (*P* = 0.25), body mass index (*P* = 0.7), height (*P* = 0.11), or weight (*P* = 0.56).

**Table 1:**
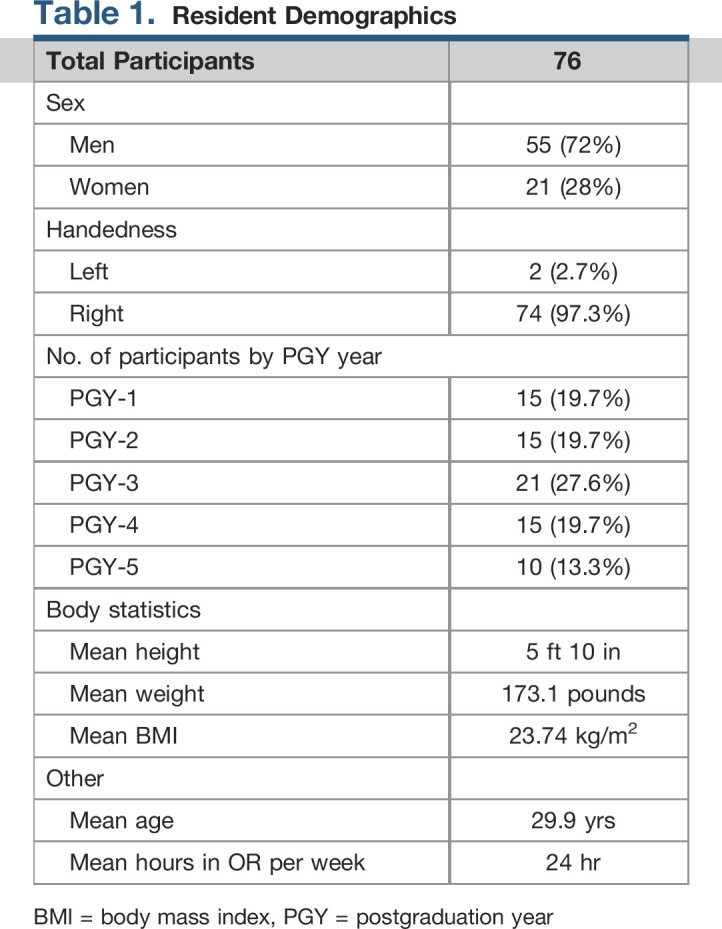
Resident Demographics

Total Participants	76
Sex	
Men	55 (72%)
Women	21 (28%)
Handedness	
Left	2 (2.7%)
Right	74 (97.3%)
No. of participants by PGY year	
PGY-1	15 (19.7%)
PGY-2	15 (19.7%)
PGY-3	21 (27.6%)
PGY-4	15 (19.7%)
PGY-5	10 (13.3%)
Body statistics	
Mean height	5 ft 10 in
Mean weight	173.1 pounds
Mean BMI	23.74 kg/m^2^
Other	
Mean age	29.9 yrs
Mean hours in OR per week	24 hr

BMI = body mass index, PGY = postgraduation year

### Symptomatology

**Figure 1:**
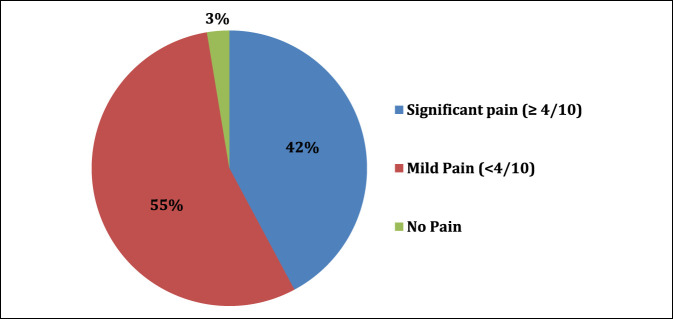
Pie chart demonstrating severity of pain scores after a full day of operating. Notable pain was termed pain ≥ 4/10 using the numeric rating scale.

**Figure 2:**
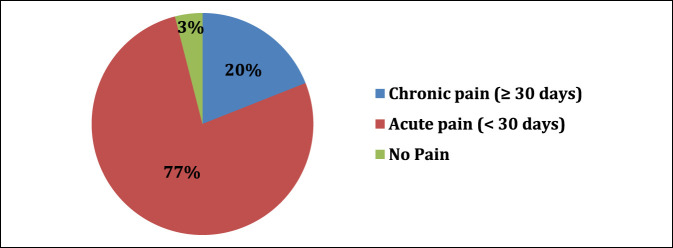
Pie chart demonstrating the chronicity procedural related pain among orthopaedic surgery residents. Chronic pain was termed pain lasting ≥ 30 days straight.

**Figure 3:**
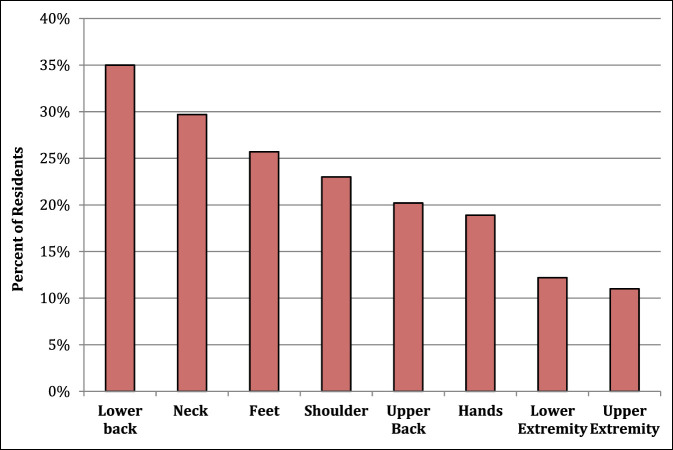
A bar graph demonstrating notable pain among orthopaedic residents stratified by anatomic location. Notable pain was termed pain ≥ 4/10 using the numeric rating scale.

In our sample, 97% of residents experience MSP after a day in the OR. Of the residents who experienced MSP, 42% experience notable pain (≥4/10 pain) (Figure 1). The average pain score after a full day of operating was 3.52 (SD 1.95). 20.2% of residents have or are currently experiencing chronic pain due to procedural-related MSP (Figure 2). Notable levels of MSP (≥4/10 pain) were found in resident surgeons, with the most frequent location being the lower back (35% of residents), followed by the neck (29.7% of residents), and finally the feet (25.7% of residents). Figure 3 shows a complete list of anatomic locations that respondents reported to have a notable level of pain (≥4/10 pain). Among residents who experience pain in a specific anatomic location, pain scores were most severe in the following locations: lower back (3.8/10), upper extremity excluding hands (3.8/10), and feet (3.5/10).

### Beliefs

Of all respondents, 34.3% of the residents feel that the MSP they experience in the OR will influence their ability to do surgical procedures in the future. Participating residents were asked to select the orthopaedic subspecialties (as many as they wanted) they felt were most associated with high MSP. Spine was the subspecialty most associated with higher MSP because it was selected by 62% of residents; this was followed by trauma (54% of residents) and adult joint reconstruction (52% of residents). In 34.3% of the residents, the MSP experienced during a specific rotation plays a notable role in deciding which fellowship they will pursue. Three-fourths (75.7%) of residents feel that using protective lead notably contributes to increased MSP while operating. Furthermore, 41.4% of residents state wearing loupes, exhaust suits, and headlamps also contributes to increased procedural-related MSP. A notable number of residents (76%) are concerned about the long-term effects of intraoperative radiation to their body, specifically their thyroid (62% of residents), eyes (60% of residents), and reproductive organs (60% of residents).

### Attitudes

Despite the high prevalence of MSP, 93% of residents were satisfied with their work at least a few times a week. In addition, 97.1% of residents would describe themselves as pretty happy to very happy. 17.4% of residents feel increased callousness at least a few times a week. Almost one-fourth (24.6%) of residents felt burnt out at least once a week. A positive correlation exists between higher pain scores and lower work satisfaction (*P* = 0.005), burnout (*P* = 0.04), and callousness toward others (*P* < 0.0001). No association exists between PGY and burnout (*P* = 0.22) or PGY and callousness (*P* = 0.13). In addition, no association exists between sex and burnout (*P* = 0.94) or sex and callousness (*P* = 0.09).

### Behaviors

**Figure 4:**
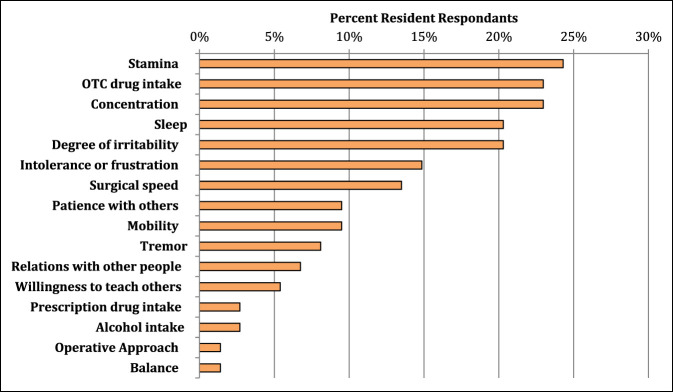
Bar chart demonstrating how procedural-related pain affects the behavior and daily life of orthopaedic surgery residents.

**Figure 5:**
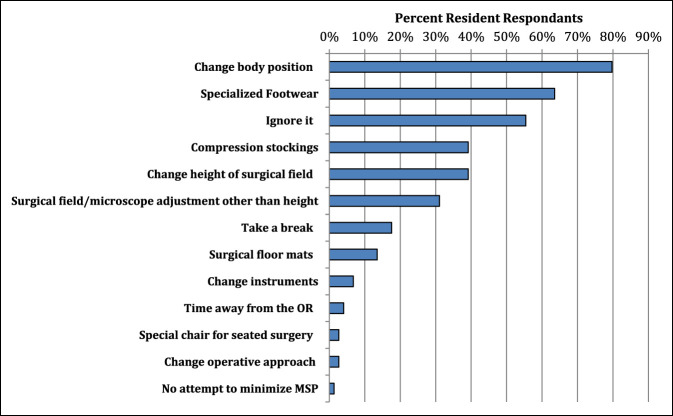
Bar chart demonstrating the most common ergonomic approaches used by orthopaedic residents to minimize procedural-related pain.

**Figure 6:**
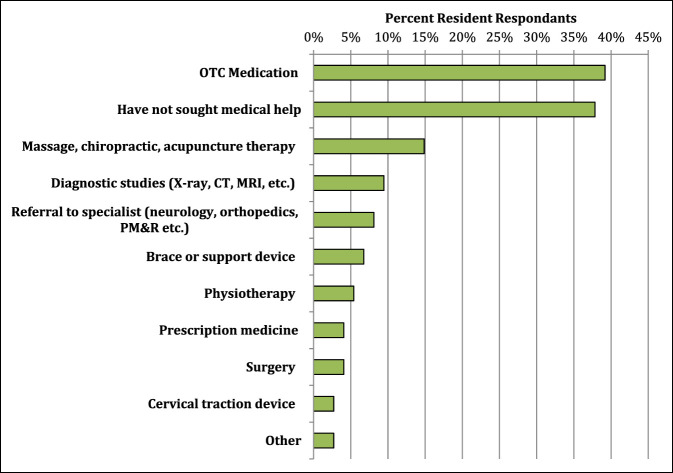
Bar chart demonstrating the most common avenues of medical care sought to address procedural-related pain among orthopaedic residents.

A wide range exists for how MSP affected the daily life of the surveyed residents. The most notable effects were on resident stamina at work, concentration, sleep, and degree of irritability, which were all significantly associated with higher MSP scores (*P* < 0.05). See Figure 4 for a full list of how procedural-related MSP affects the daily life residents. Several techniques were used in the OR by residents to minimize procedural MSP. Among the most common intraoperative techniques used were intraoperative positional changes, specialized footwear, compression stockings, or changing working height using either a step stool or a chair to sit down. Figure 5 displays a full list of ergonomic techniques residents used to decrease procedural-related MSP. Surprisingly, an association was noted between higher numbers of different ergonomic techniques used in the OR and higher pain scores (*P* = 0.018). Once with MSP, most residents (62%) are proactive in seeking medical help for procedural-related MSP. Seeking medical help for the pain is notably associated with higher pain scores (*P* < 0.001). The most common medical sources used to help alleviate MSP were over-the-counter (OTC) medications, massage therapy, diagnostic imaging, and referral to medical specialists (Figure 6).

## Discussion

Orthopaedic surgeons experience numerous occupational hazards. Previous studies have shown the impact these exposures can have on the practicing surgeon.^2,4,5,11,13,14^ However, a paucity of data exists in the literature regarding the effects of these hazards on resident physicians. To the authors' knowledge, this is the first study to report the prevalence of MSP and characterize its effects on orthopaedic surgery residents from multiple random institutions. One of the key findings in this study is the extremely high rate of orthopaedic residents who experience MSP after a day in the OR (97%). In comparison, a 2015 study of MSP among construction workers demonstrated only 50% experienced MSP.^20^ Furthermore, 42% of residents had pain rated greater than or equal to 4/10. To further put this into perspective, a score of 4 on the numeric rating scale is considered “moderate” and has a likelihood ratio of causing clinically important pain of 2.7.^21^ Evidence suggests that a value of 4/10 represents the tolerable pain threshold when evaluating patients in a postoperative setting.^22^ In fact, some orthopaedic postoperative pain control regimens permit the use of narcotic pain medication when patients experience pain of 4/10 or greater after surgery. After a full day of procedures, almost half of all surveyed residents (42%) would qualify for a dose of narcotic pain medication under these institution-specific postoperative pain control regimens.

Many of our findings correlate with previous studies in populations of attending surgeons. Stucky et al^10^ did a systematic review and meta-analysis of surgical ergonomics and found the two most common locations of MSP to be back pain, followed by neck pain, which align with our results. These symptoms are likely due to strain during prolonged retraction, holding extremities, or looking down into the operating field for a prolonged period of time. Orthopaedic residents may be at higher risk of poor ergonomic positioning in the operating field because they often assume an assistant position and must accommodate to obtain a clear view of the surgical field. This could be especially true in cases with limited exposure including spine surgery and total hip arthroplasty. Another possible contributor to MSP, especially in the neck and back, is the use of lead, loupes, and other protective equipment. Of our respondents, 75.7% felt that the use of protective lead contributed notably to MSP, and 41.4% feel that loupes, exhaust suits, and headlamps also notably contributes to higher MSP. Depending on the brand and type, lead aprons can weigh from 5 to 17 pounds. Numerous orthopaedic procedures require lead protection while using intraoperative fluoroscopy, and many cases can take several hours. This extra weight on the shoulder, head, and neck undoubtedly leads to increased MSP. Although cumbersome, these accessories are essential either to aid in surgery (loupes and headlamps) or to protect the resident surgeons (lead and exhaust suits). Orthopaedic residents are aware of the known risks of radiation exposure^2^ because 76% of respondents reported concern of the long-term effects.

Interestingly, despite the high rates of MSP, orthopaedic residents report a very high rate of overall satisfaction most days per week (93%). We found statistically notable associations with high MSP scores and lower work satisfaction (*P* = 0.005), burnout (*P* = 0.04), and callousness toward others (*P* < 0.0001). This is a concerning finding at an early stage in a trainee's career. With such a high overall rate of MSP, we would expect many of these respondents to have continued MSP on completion of training, which has been demonstrated in several other surgical specialties.^4,6,9,10,12^ Higher pain scores over a long period of time could lead to increased, burnout, callousness, and lower satisfaction overall. This is a concern for the physical and mental health of the surgeons and the potential health of the workforce overall.

As awareness of procedure-related MSP has grown, increased attention has been paid to assessing current education initiatives and to generating solutions to the problem. At present, few surgical training programs provide any type of surgical ergonomics (formal or informal) education (SEE), and far fewer programs offer formal SEE. This is often because of a lack of evidence-based educational models and lack of certified staff.^23^ Despite a lack of formal ergonomics education in surgical specialties, some preliminary guidelines have been suggested to ease the ergonomic burden of surgeons, including the use of tools, monitors, and body position.^24^ In an attempt to address MSP among surgeons, many surgical departments across the country have implemented ergonomic programs. These programs have implemented numerous alterations to decrease the incidence of MSP among their surgeons. Some of the modifications implemented include the use of loupes, specialized footwear, and exoskeletons to improve posture in surgeons.^25,26^ Table height calculations based on standing elbow height have also been shown to potentially improve operator posture.^27^ Although alterations to technology have promising potential for improving surgeon MSP, the results are mixed across interventions. Alternatively, behavioral changes, such as surgeon “microbreaks” and resistance training, show promise across multiple studies.^28,29^ Despite proposed solutions and guidelines, surgeon discomfort remains highly prevalent. Some have cited institutional culture as a primary reason for underreporting injuries, whereas many physicians are unaware of guidelines for improved ergonomics.^16,25,30^ Addressing the knowledge and education gaps pertaining to surgeon MSP, in addition to increased institutional support, will be vital to reducing surgeon pain and discomfort, preserving productivity, and increasing career longevity.

Addressing MSP in surgical residents is crucial to decreasing chronic pain that could ultimately impact career longevity. This is not only important for the individual resident and program but also for society as a whole because demand for orthopaedic surgeons in the United States continues to grow.^31^ It is challenging to quantify the total societal costs of surgeon MSP because of underreporting and the culture of physician resilience; however, several studies relate that missed work and reduced productivity may have financial impacts on the affected surgeon while also placing increased burden and caseload on their colleagues.^32,33^ Forming good ergonomic habits early in one's career may prevent the development of MSP later in life. We hope that this study raises awareness to this problem and ultimately serves as a reminder to both trainees and practicing orthopaedic surgeons of the potential impact of poor surgical ergonomics.

This study has several limitations. First, we had a limited number of respondents with 76 residents, despite submitting the survey to 78 PDs. This introduces potential selection bias, and perhaps, this sample is not completely representative of the entire American College of Graduate Medical Education orthopaedic residency cohort. In addition, surveys lend themselves to volunteer response bias and nonresponse bias. We recognize that respondents to this survey may have been interested in reporting their experiences because of their preexisting MSP. In addition, an increased percentage of female respondents were noted above the average of women in orthopaedic surgery residencies^34^ which could represent a potential source of bias. For this purpose, a separate analysis was did, which did not demonstrate any notable sex correlations with survey responses. We attempted to write neutral questions in our survey to minimize the risk of any leading questions that would bias the readers. The authors were as explicit as possible in that all injuries to be reported as part of the survey were orthopaedic work-related injuries. It is likely that some of the pain reported in the survey may not have its genesis directly from procedural related discomfort, but instead could represent preexisting conditions that were flared up by orthopaedic procedural work. Regardless, orthopaedic procedural related work would represent a direct contributor to their current pain. The association between physical pain and psychological symptoms has been well studied.^35^ The possibility exists that some MSP among residents could be a result of dissatisfaction/burnout manifesting as a physical symptom.^36^ See Appendix A for the administered survey, http://links.lww.com/JG9/A114. As mentioned previously, it is difficult assess surgeon MSP because of underreporting and the culture of physician resilience, which could additionally play a role in survey responses.^17^

Future studies should look to analyze surgical posture and ergonomics in orthopaedic residents. The results of these studies can help inform and create training programs to emphasize appropriate intraoperative posture and increase awareness for compromising positions. Ideally, a set of guidelines could be developed, reviewed, and endorsed by the accrediting bodies for surgical residents and specific orthopaedic societies to address this problem.

## Conclusion

The prevalence of acute and chronic MSP among orthopaedic residents is extremely high. Our study demonstrates that MSP has a notable impact on resident stamina, concentration, degree of irritability, and other burnout symptoms. The results of this study highlight the importance of limiting compromising procedural positions, ergonomic optimization, and increasing the awareness of the importance of ergonomics among residents. This could have future implications on productivity and career longevity.
